# Characterization of Bonding between Asphalt Concrete Layer under Water and Salt Erosion

**DOI:** 10.3390/ma12193055

**Published:** 2019-09-20

**Authors:** Wuping Ran, Yu Zhang, Ling Li, Xizhong Shen, Hailin Zhu, Yongbo Zhang

**Affiliations:** 1 School of Civil Engineering &Architecture, Xinjiang University, Urumqi 830047, China; ranwp@xju.edu.cn (W.R.); lil@xju.edu.cn (L.L.); superwill007@126.com (X.S.); seamouse2580@163.com (H.Z.); xjzhangyb@163.com (Y.Z.); 2 Yellow River Institute of Hydraulic Research, Yellow River Conservancy Commission, Shunhe Road No. 45, Zhengzhou 450003, China

**Keywords:** road engineering, bonding condition, shear test, numerical simulation, shear modulus, friction coefficient

## Abstract

The contact state between layers of asphalt pavement not only has a significant effect on the mechanical response of road structure but is also the bottleneck of research on the mechanical behavior of pavement structure at present. In this paper, the effects and laws of different water–salt entry modes, salt solution concentrations, and temperatures coupling on the contact state between base and surface layers are studied by a 45° inclined shear test. The simulation and verification of each working condition are carried out by ABAQUS (Dassault, Paris, France) the friction coefficient between layers is reversed, and the actual contact state between layers is characterized in order to realize comprehensive evaluation and reasonable expression. The results show that different modes have different effects on contact characteristics. At the same temperature and concentration of the salt solution, bonding of water and salt erosion is the best, followed by direct erosion, with the worst being from bottom to top, and the interlayer bonding condition is weakened with increase in temperature. The relative accuracy of the software simulation and test analysis was as high as 92% and the friction coefficient of the water-free salt erosion test piece was found to be about 0.85 at 25 °C, while after the bottom-up erosion of the 14% salt solution the friction coefficient was found to be about 0.43, which indicates that the corrosion of the water–salt will have a great effect on the bonding condition between the structural layers of the road.

## 1. Introduction

Asphalt concrete pavement is the most commonly used road structure of highways at all levels [1], and has the advantages of being energy saving and emissions reducing, being low cost, having convenient maintenance, and so on [2]. The pavement is composed of multiple layers affected by a combination of traffic load and climate change [3], and poor interlayer bonding of asphalt pavement will lead to structural problems, such as slippage, delamination, and top-down cracking [4]. The continuous evolution and deterioration of the contact state between layers are the main reasons for aggravating the development of fatigue damage and permanent deformation of asphalt pavement [5]. Therefore, it is important to pay more and more attention to the contact state between layers in the whole life cycle of asphalt pavement.

The concept of the influence of interlaminar contact state on pavement was first put forward at the first International Conference on Asphalt Pavement Structure Design in 1962. Livneh suggested that the change of the contact state between layers may affect the stress distribution of asphalt pavement and that a decrease in bonding force will affect the bearing capacity of asphalt pavement [6]. The first study of interlaminar bonding was by Uzan [7]. The results showed that the stress and strain of pavement structure are greatly affected by a different bonding degree in the process of the interlaminar bonding state from complete continuity to complete sliding. Since then, road researchers have begun to pay attention to the interlayer contact of road structures. Several research studies have confirmed that proper knowledge of interlayer bonding conditions is imperative to accurately evaluate the service life of pavement [8,9]. In the service life and maintenance of pavement, proper bonding between layers is one of the most important factors, and Aposeiras et al. found that the most important factor affecting the contact between layers is the macrostructure of the surface and the amount of the bonding layer, while the effect of the type of bonding agent can be neglected [10,11]. Tashman et al. studied the influence of several construction methods on the interfacial bond strength between rolled and rolled sections [12]. West et al. evaluated the effects of type, dosage, mixture type, test temperature, and atmospheric pressure of bonding layer on bonding strength through laboratory tests [13]. Liu et al. studied four types of interfaces: no gap, no adhesive layer, no gap but an adhesive layer, no adhesive layer but a gap, and an adhesive layer. The shear test was carried out at room temperature by a self-made interface shear instrument [14]. Tarr et al. carried out full-scale load-induced strain and drop-weight deflectometer tests to measure the bond degree between Portland cement concrete pavement and lean concrete base (LCB) prepared by five common interface treatment methods [15].

Saline soil is widely distributed and the climate is complex in saline soil areas, which has a significant impact on pavement structure and especially on the bonding condition between layers of asphalt pavement. Because of the complex climate and serious salinization, there are many diseases in these areas, and therefore, how to solve the contact state between asphalt pavement layers under the influence of salinization has become an urgent academic problem for road construction and scientific researchers. Salinization erosion of road structure begins with salt invasion caused by saline soil foundation or filling, natural environment, and human factors. The main manifestations are as follows: firstly, water and salt migrate from bottom to top, gradually eroding the bonding characteristics of pavement structure layers; secondly, in the seasonally frozen zone, water and salt migrate from top to bottom due to the use of ice and snow solvents, gradually eroding the bonding characteristics of the road structure. At present, research on the damage of asphalt pavement caused by water and salt mainly focuses on two aspects: deicing salt in frozen areas and damage to physical and mechanical properties of asphalt mixtures by salt fog environments in coastal areas. The salt concentration in the pavement can cause damage to the pavement [16]. The surface damage, weight loss, and splitting tensile strength loss of ordinary concrete and steel-fiber-reinforced concrete is different after different freeze–thaw cycles in water and sodium chloride solution [17]. Yang et al. used ultrasonic technology to study the damage of concrete specimens caused by different salt erosion [18]. Hassan et al. compared the destructive effects of different salts on the durability of aggregate and asphalt concrete for pavement construction under freeze–thaw cycles [19]. Cui et al. tested three conventional indexes of base asphalt, modified asphalt and rubber-powder-modified asphalt before and after salt freezing and analyzed the factors affecting the high-temperature and low-temperature performance of asphalt by range analysis method and grey relational entropy method. [20] Liu et al. studied the low-temperature performance and salt release characteristics of anti-freezing asphalt concrete (AFAC) under different moisture conditions. Hence, a low-temperature bending test and an electrical conductivity test were carried out to evaluate the performance of anti-freezing asphalt concrete [21]. The effects of water, acid, alkali, and salt solution on the properties of asphalt mixture and SBS-modified asphalt mixture can be seen via the freeze–thaw cycle test, the void ratio test, the Rotman test and the indoor test [22]. Ketcheson et al. studied the effect of road salt (sodium chloride) on water and chloride movement in pervious concrete structures in a laboratory environment [23]. Nili et al. evaluated the salt resistance of long and short freezing period samples under freeze–thaw cycles [24]. Throughout the current research on the state of layer-to-layer contact there is a main focus on influencing factors. Although these factors have strong applicability, they are not representative of different geographical environments. Because of the complex climate and serious salinization present in many areas, many diseases of asphalt pavements appear under the special service environment of water, temperature, and salinization. Thus, how to solve the contact state between asphalt pavement layers under the influence of salinization has become an urgent academic and engineering problem for road construction and scientific researchers.

It can be seen that the change and trend of asphalt pavement interlayers influenced by various factors can be obtained by tests, but it is difficult to judge how much the influence of various factors is. Therefore, how to combine experiments with theoretical analysis and software simulation, from qualitative analysis to deeper quantitative analysis, has become a problem that needs further consideration. With this in mind, the contact state characteristics and laws of asphalt pavement base layers under water and salt erosion are characterized in this study and on this basis, interlayer contact is analyzed by numerical simulation.

## 2. Experimental Program

### 2.1. Materials and Methods

Five centimeter cement-stabilized macadam layers and 5 cm asphalt concrete layer composite rutting slabs were formed, these being 300 mm long, 300 mm wide, and 10 mm thick. The cement-stabilized macadam layer (5 cm thick) was first formed, after which emulsified asphalt permeable oil was sprayed on it and an asphalt concrete rutting layer (5 cm thick) was formed on the cement-stabilized macadam layer. The layered construction rolling process was simulated. The amount of permeable oil sprayed was 0.4 L/m^2^ [25]. After the forming of the composite rutting slab was completed, the 15 mm parts of each edge of the composite rutting slab were cut off by a cutting machine and the remaining parts were cut into 90 mm long, 90 mm wide, and 100 mm high specimens. Each rutting slab was able to form nine specimens. The direct erosion of water and salt was to form a cement-stabilized macadam layer and then salt solution was to be sprayed. The spraying amount was 120 mL, which was determined by many experiments and calculation analysis. In order to ensure the uniform distribution of salt on the surface of the specimen, three sprays were successively sprayed. Finally, the permeable oil was sprayed and the asphalt concrete layer was formed on top of it to complete the production of the whole specimen.

**Figure 1:**
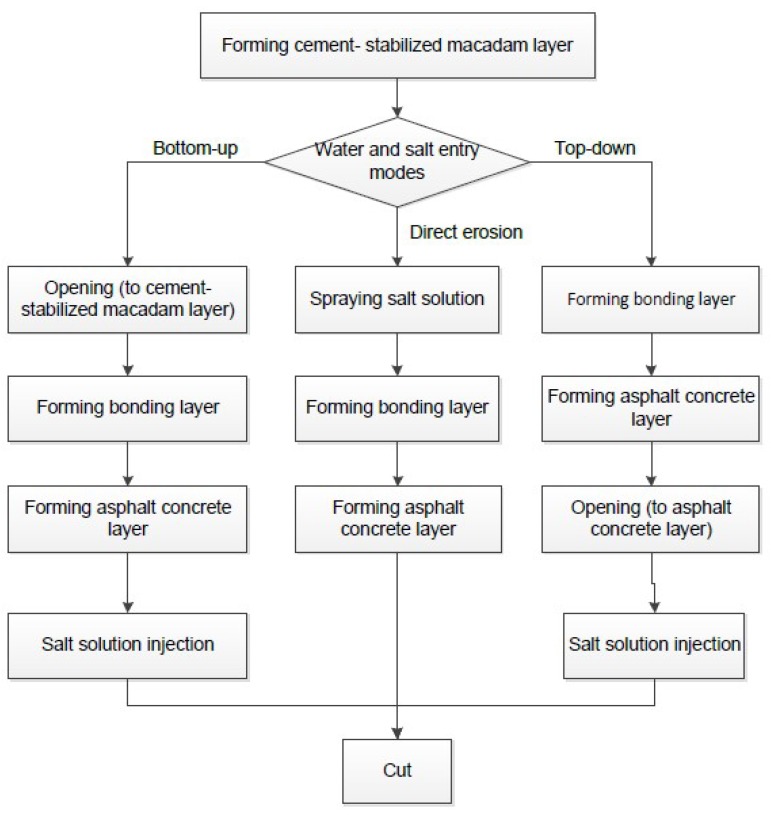
Flow chart of specimen forming.

When water and salt eroded specimens from bottom to top after the water-stabilized layer was made, holes were nailed out with steel nails with a diameter of 2 mm and length of 50 mm in the cement-stabilized macadam layer, and the holes ran through the whole cement-stabilized macadam layer. The pavement of permeable soil and asphalt concrete surface was able to be continued after the maintenance of the cement-stabilized macadam layer was completed. The steel nails were pulled out and a stainless steel hollow circular pipe with an outer diameter of 2 mm and a length of 50 mm were inserted into the hole. The rutting plate was placed face down and flat on the floor. The salt solution was injected into the hollow tube with a 0.1 mL calibration unit injector according to the prescribed dosage. Considering the maximum spraying amount of 120 mL on the surface of the 300 mm by 300 mm specimens for direct erosion between layers, the reference value was 3.5 mL per injection in each hollow tube, and the hollow tube orifice was sealed immediately after the injection was completed. After the solution was completely immersed, the solution was injected again and completed three times. When water and salt eroded the specimens from top to bottom, the holes were nailed out on the asphalt concrete surface and hollow pipes inserted into the holes. After curing, the salt solution was injected three times to complete the simulation test of water and salt erosion. The forming process of three different water and salt entry modes as detailed in Figure 1.

According to the modes of salt entrance and the factors affecting the interlayer performance of road structure, three influencing factors were selected in this paper, namely, water and salt entry mode, chloride solution concentration, and temperature. Considering the water and salt sources and migration modes between corroded pavement layers, the top-down and bottom-up water and salt entry modes were determined. In addition, in order to compare the erosion degree between layers caused by different entry modes of water and salt, direct erosion between layers was also considered to simulate the maximum accumulation state of salt between layers. Chen Ma suggests that the concentration of chlorine salt snow-melting agent should not exceed 14% in road snow-removal projects, a conclusion that was determined by studying the influence of chlorine salt snow-melting agent concentration on asphalt and mixture performance [26]. According to the classification standard of saline soil and the industry standard of road deicing and snow removal in China, the chloride concentrations in this experiment were determined to be 0%, 7%, and 14% by mass concentration. At the same time, 10 °C, 25 °C, and 40 °C were selected to carry out the tests, respectively. Before the shear test, the specimens were placed together with the test model in a constant greenhouse which had reached the test temperature, and the heat preservation time was not less than 5 h. Parallel tests were conducted for each group of three specimens.

### 2.2. Test Device

The inclined shear test was carried out using a computer-controlled electro-hydraulic servo universal testing machine and a self-designed jaw. In order to reduce the influence of bottom friction on the test results, a smooth circular steel bar coated with lubricant was placed between the bottom clamp and the bottom plate of the test machine [27]. The bonding force between layers of the asphalt pavement structure was relatively small. Strain control was chosen. Combined with the fact that the shear strength between layers was relatively small, a loading rate of 5 mm/min was adopted for this paper.

### 2.3. Shear Test Calculation

The interlayer shear strength was calculated by the following formula:

(1) $$\sigma_{n}=\frac{P}{A}(\cos\alpha +f\sin\alpha ),\;\tau_{n}=\frac{P}{A}(\cos\alpha -f\sin\alpha )$$

where *σ_n_* is normal stress, *τ_n_* is shear stress, *P* is the applied load, *α* is the fixture inclination angle (45° in this paper), and *f* is the rolling friction coefficient (0.002–0.004, generally 0.003) [28].

**Figure 2:**
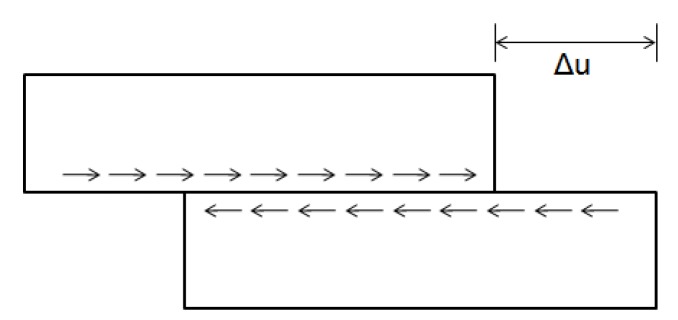
Goodman model schematic diagram.

The contact state between layers was analyzed by the Goodman model. According to Goodman, when the relative horizontal displacement *u* occurs between the upper and lower layers of the pavement, the shear stress of the interlayer interface can be expressed as follows. The schematic diagram of the model as shown in Figure 2.

(2) $$\tau_{n}=K\cdot \Delta u$$

where *K* is the interlaminar shear modulus and ∆*u* is the relative displacement at the interlayer interface.

### 2.4. Numerical Analysis

#### 2.4.1. Establishment of Model

In the process of forming the specimens, specimens with different contact states between layers were formed by setting different water and salt entry modes, salt solution concentration, and temperature conditions. However, through the oblique shear test, we were only able to obtain the interlayer shear modulus (K) to reflect the change and trend of the contact range affected by various factors, via which it is very difficult to judge how good or bad the contact state between layers is. Hence, how to correspond the actual test with theoretical analysis, from qualitative analysis to deeper quantitative analysis, became a problem that needed further consideration in the analysis. In the oblique shear test, we first obtained the established contact state between layers by means of the test, and then we used the press to conduct the oblique shear test on the specimens to measure the relative displacement between the base and the surface layer and the maximum strength. Conversely, we used ABAQUS to calculate the interlayer friction coefficient between the base layer and the surface layer and to calculate the relative displacement between the base layer and the surface layer under the action of the known force (the force obtained from the test). A relative displacement was obtained for each given friction coefficient. When the calculated relative displacement was the closest to the experimental displacement, it was assumed that the calculated interlayer friction coefficient can be used as the representative value of the interlayer contact state under real conditions in the test.

**Figure 3:**
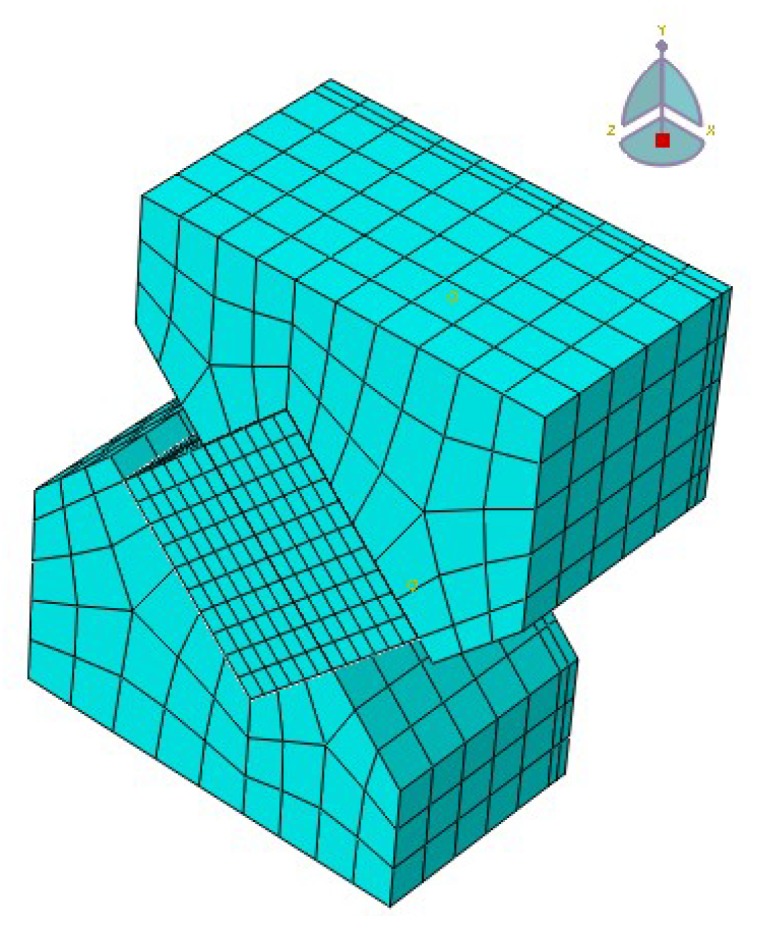
Schematic geometry of the model.

ABAQUS was used to build the model, as shown in Figure 3. The dimensions of the specimen and die in the finite element software were identical to those in the actual test at 25 °C. The shear angle was 45° and the material parameters of each layer in the model are detailed in Table 1. The load applied in the software was the real load value obtained from the test. By changing the friction coefficient of the contact surface, the displacement was calculated and compared with the displacement obtained from the test. The friction coefficient of the base–surface interface which was close to the actual working condition was obtained.

#### 2.4.2. Realization of Contact Simulation in ABAQUS

Composite pavement is a multi-layer structure system and the contact state between layers has a significant impact on the mechanical response. One of the most direct ways to analyze contact problems by using the finite element method is to set up a special type of element on the contact surface which is commonly called the contact element but is also known as the interface element. The contact behavior between two surfaces includes three relationships: continuous, semi-continuous, and smooth. In ABAQUS, "constraint" can be used to simulate the continuous state of two contact surfaces. For the semi-continuous contact state between two contact surfaces, it is generally expressed by imposing the "friction coefficient" between the contact surfaces. The larger the friction coefficient, the better the contact state. When the friction coefficient is zero, the contact surface is completely smooth. In fact, the layer-to-layer contact of the pavement structure is neither completely continuous nor completely smooth but between the two extreme cases. In the simulation of ABAQUS contact problems it is necessary to define the interaction of the contact surfaces. The interaction of contact surfaces consists of two parts, namely, normal and tangential, where the tangential action includes the relative motion of the contact surface and the possible frictional shear stress. When the shear stress at the interlayer interface is less than the shear strength at the interlayer interface, there is no relative movement between the layers; when the shear stress at the interlayer interface is greater than the critical shear strength at the interface, relative movement between the two layers may occur.

## 3. Results

### 3.1. Test Model

**Figure 4:**
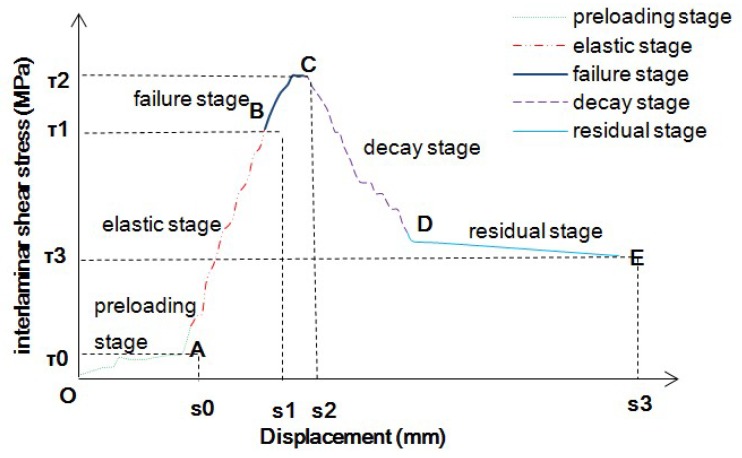
Four interlaminar shear stress and displacement curves.

The curves of the interlaminar shear stress-displacement obtained from the experiment show the same change rule: the interlaminar shear stress-displacement curves roughly experience four stages in the 45° shear test (the preloading stage in the figure is a "preloading" and "stabilization" stage, which was not included in the scope of this study, and the interlaminar shear stress value at the end of this stage is *τ_0_* and the corresponding interlaminar relative slip displacement is *s_0_*). As shown in Figure 4, the curvature of the curve was found to be equal to the corresponding interlaminar shear modulus of the base surface.

The elastic stage (the AB stage) is the preliminary loading stage in which stress is almost linear with displacement. There was no obvious crack around the specimen. The shear strength at the endpoint (point B) of the elastic stage is expressed by *τ_1_* and the relative sliding displacement between layers is expressed by *s_1_*. The failure stage (the BC stage) is similar to the yield stage of the material and the curvature of the curve decreases gradually in this stage compared with the first stage but the stress continues to increase with the displacement and ultimately reaches the maximum shear stress (point C ultimate shear strength is denoted as *τ_2_* and its corresponding relative sliding displacement between layers is denoted as *s_2_*). It was considered that the base–surface layer had been destroyed at this stage. At this stage, obvious cracks appeared in the specimens which gradually developed and extended to the surrounding area. In the decay stage (the CD stage), the interlaminar shear stress decreases rapidly due to interlaminar failure and the interlaminar interface structure of the formed specimens has been destroyed. In the residual stage, the interlaminar shear stress does not change significantly with the displacement but basically remains at the level of residual strength. The residual stage point E shear strength is expressed by *τ_3_* and the corresponding interlayer relative sliding displacement is expressed by *s_3_*. The residual strength (*τ_3_*) in Figure 4 was found to be about 45% of the ultimate shear strength (*τ_2_*). The peak value of shear stress (*τ_2_*) was observed to be the maximum shear stress that can be endured when the interlaminar shear failure occurs. It represents interlaminar cohesion and friction. The larger the value of *τ_2_*, the better the interlaminar shear performance was found to be. The larger the relative sliding displacement *s_2_* corresponding to the peak value, the better the interlaminar cohesion performance was observed as [29]. Residual strength *τ_3_* was the shear strength that specimens can bear after shear failure. The larger *τ_3_* was, the stronger the deformation recovery ability after failure was found to be.

The shear strength of the interface between the asphalt concrete layer depended on the bonding performance between them. In the course of the slip, the bonding force of the interlayer interface was mainly composed of the asphalt bonding force between composite layers and the chemical bonding force on the concrete surface, the mechanical bonding force of the rough interface between two different types of materials and the friction force of two different types of concrete interface.

In the elastic stage, the shear stress mainly overcomes the interlaminar bonding force and occlusion force and the shear stress increases from 0 to the maximum. With the increase in interlaminar shear stress, the interlaminar shear stress gradually loses cohesion and the specimen slides. In the failure stage, with the further increase in interlaminar shear stress, the mechanical occlusion force of two different types of materials with a rough interface begin to play a role gradually. When the mechanical occlusion force and bonding force reach the maximum value (corresponding to the peak value of interlaminar shear stress in the figure), the specimen reaches shear failure. In the attenuation stage, when the shear stress reaches its peak value, the specimen slips relatively, the interfacial bonding force and mechanical occlusion force disappear mostly, and the shear stress decreases sharply. At this time, the bonding force of the interlayer interface was found to be mainly the friction force caused by relative displacement between layers. When the specimen reached the maximum shear stress, destructive instability occurred, and the displacement increased while the shear stress decreased continuously until it reached the final residual strength. In the residual stage, the shear stress overcame the friction caused by the interlaminar displacement. The increase in displacement had little effect on the shear stress and there was no interaction between the interlaminar interfaces. Therefore, it was reasonable and feasible to use an interlayer shear modulus to evaluate the contact state of the pavement subgrade.

### 3.2. Effect of Different Factors on Interlayer Contact State

#### 3.2.1. Water and Salt Entry Mode

**Figure 5:**
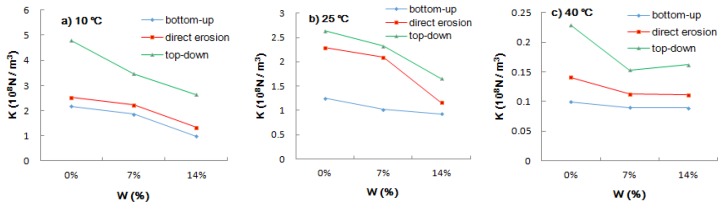
Effect of water and salt entry mode on interlaminar shear modulus: (**a**) 10 °C; (**b**) 25 °C; (**c**) 40 °C.

Figure 5 shows that at the same temperature and concentration of the salt solution, the interlaminar shear modulus values obtained under the three entry modes were in the order of top-down > direct erosion > bottom-up. It shows that the top-down mode had the least influence on the contact state between layers in comparison to the other two modes. At this time, the interlayer bonding was best, followed by the direct erosion mode, and the bottom-up mode was the worst. The interlaminar shear modulus curves of the three different salt solutions were found to be nearly parallel at 10 °C. The interlaminar shear modulus can be seen to decrease in a positive proportion with the increase in salt solution concentration and the change in the interlaminar shear modulus of salt solution concentration from 7% to 14% is obviously greater than that from 0% to 7%. The interlaminar shear modulus at 0% and 7% concentration changed in a positive proportion at 25 °C but the change in interlaminar shear modulus from bottom-up to direct erosion was steeper than that at 10 °C, while the change from direct erosion to top-down was slower than that at 10 °C and the interlaminar shear modulus at 14% concentration was much lower than the "expected value" under the trend of positive proportion change. Different concentrations had little effect on the bottom-up mode but were found to be highly sensitive to the direct erosion and top-down modes. Under direct erosion, the interlaminar shear modulus was observed to decrease by 8.75% from 0% to 7% of the salt solution concentration, while the interlaminar shear modulus decreased by 44.59% from 7% to 14%. The interlaminar shear modulus was found to vary slightly from 7% to 14% for three different water and salt entry modes at 40 °C. The bottom-up and direct erosion values can be seen to be almost the same and the top-down ratio of 14% was only 5.67% higher than that of 7%.

The position of the water–salt film formed by injection of water–salt in different water–salt entry modes (specimen forming mode) was different. It can be assumed that the water–salt injection from bottom to top was between the cement-stabilized macadam layer and the bonding layer while that from top to bottom was between the bonding layer and asphalt concrete surface layer and the influence of a cement-stabilized macadam base and asphalt concrete surface layer on water–salt was also different.

Generally, there were polar groups on the stone surface which were able to form a short bond with water molecules through a hydrogen bond and so had a great affinity for water. Macroscopically, the material surface was wetted by water. On the other hand, the asphalt surface had non-polar groups (containing a C–H bond). The attraction between water molecules and asphalt molecules was much less than the cohesion between water molecules. Macroscopically, this showed that the material surface was not wetted by water [30]. After the asphalt mixture is immersed in chloride solution the salt solution penetrates into the asphalt mixture through the void and the chloride salt emulsifies the asphalt, which greatly reduces the cementation performance between the asphalt and aggregate. The cohesion of asphalt decreases and the shear strength of the asphalt mixture decreases. Under the action of a chloride snow-melting agent, the asphalt composition will change, the saturated fragrance and aromatic content will decrease, the quantity of asphaltene and gum will increase, the high-temperature performance of asphalt will be improved, the asphalt will become hard, brittle, and flexible, and the interaction between asphalt and aggregate will be weakened [26].

#### 3.2.2. Salt Solution Concentration

**Figure 6:**
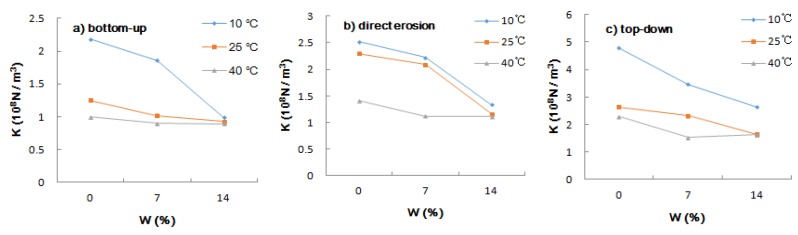
Effect of salt solution concentration on interlaminar shear modulus: (**a**) bottom-up; (**b**) direct erosion; (**c**) top-down.

Figure 6 shows the influence of the salt solution concentration on the interlaminar shear modulus. It can be seen that the trend of the interlaminar shear modulus was similar given three ways of salt solution entry. At 40 °C, the effect of salt solution concentration on the interlaminar shear modulus was obviously weaker than at 10 °C and 25 °C. The interlaminar shear modulus decreased by 10.51%, 21.03%, and 29.09%, respectively, under the three erosion modes of salt solution concentration from 0% to 14%. The range of significant change was from 0% to 7%.

#### 3.2.3. Temperature

The interlaminar shear modulus decreased with the increase in temperature under the three water and salt entry modes. As a viscoelastic material, asphalt has special rheological properties. At high temperature, asphalt will transform from elastomer to plasticity, the modulus of stiffness will be greatly reduced, and the deformation resistance will be sharply reduced. The shear strength of asphalt at a high temperature is weaker than that at normal temperature. Because the modulus of the asphalt surface layer is different from that of the concrete layer, the compatibility of deformation is poor when the volume expands and shrinks. Under the action of high temperature, the modulus of the stiffness of asphalt decreases and cohesion and shear strength also decrease. Slipping, cracking, and deformation are easily caused by the lack of shear resistance between the asphalt layer, especially between the base and surface layers. Scholars have studied the permeation rate of water in asphalt membranes with different thicknesses at different temperatures. The results show that the higher the temperature is, the faster the water permeation rate is [31]. Asphalt is a typical temperature-sensitive material. Synchronized gravel seal was used as the middle layer of the pavement structure. The shear strength of the interlayer interface at room temperature (25 °C) and at high temperature (60 °C) was measured and compared. The results show that the interfacial shear strength at high temperature is only about 8% of that at normal temperature, which shows that the interfacial strength is highly sensitive to temperature [32].

**Figure 7:**
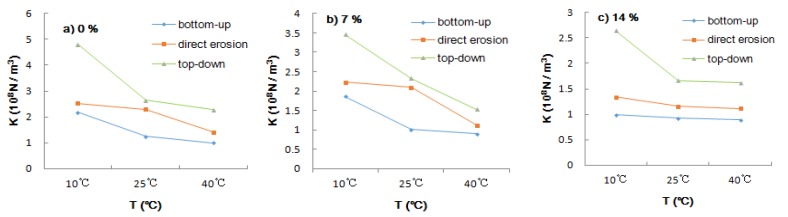
Effect of temperature on interlaminar shear modulus: (**a**) 0%; (**b**) 7%; (**c**) 14%.

As can be seen from Figure 7:

(1) At low temperature (10 °C) the interlayer shear modulus provided by the top-down entry mode is significantly better than that provided by the direct erosion and bottom-up modes and there is little difference between the direct erosion and bottom-up modes; at high temperature (40 °C) the difference between the three modes is significantly reduced.
(2) Because asphalt is a thermosensitive material, the shear modulus between the base and surface decreases rapidly with the increase in temperature. Under the same salt solution concentration and water–salt entry mode, the interlaminar shear modulus of the base–surface decreases rapidly with the increase in temperature. The attenuation rates from 25 °C to 10 °C and from 40 °C to 25 °C are as high as 45.40% and 46.34%. However, when the concentration of water and salt is 14%, the change of shear modulus with temperature under the three water and salt modes is obviously slower than that under the concentrations of the salt solution of 0% and 7%.

### 3.3. Numerical Simulation Results

In Table 2, the vertical pressure and test vertical displacement are measured directly in the oblique shear test by a servo press, which was transformed into the vertical pressure and the tangential displacement of the loading surface, respectively. In the numerical simulation calculation, a trial calculation of the interlayer friction coefficient was carried out to calculate the relative displacement between the base and surface under different interlayer friction coefficient values. The tangential displacement after deformation was measured directly and compared with the equivalent tangential displacement of the test. When the calculated relative displacement was the closest to the experimental displacement, it was assumed that the calculated interlayer friction coefficient could be used as the representative value of the interlayer contact state under the real condition in the test.

From Table 2, it can be seen that the interlayer friction coefficient under 0% salt solution erosion was significantly higher than that under 7% and 14% salt solution concentrations and that the interlayer friction coefficient decreases with increase in salt solution concentration, i.e., the higher the salt solution concentration, the worse the interlayer bonding state. At the same concentration, the top-down interlayer friction coefficient was the highest, followed by the direct erosion friction coefficient, and the bottom-up friction coefficient was the worst.

It should be pointed out that although experimental studies have proved that asphalt has strong rheological properties, these rheological problems are not considered in the numerical simulation. The rheological properties of asphalt are mainly reflected in the relationships between stress, deformation, loading time, and temperature. If the physical characteristics of the mechanical behavior of asphalt pavement structure are studied, the rheological physical properties of materials will have a significant impact on the structural mechanical behavior. However, this article mainly reflects the mechanical behavior between the base and the surface layer and considers the room temperature condition in the finite element method. Currently, it is considered that the viscous property is more remarkable than the physical property of rheology. Therefore, only the damping coefficient is considered in the numerical simulation analysis, and the rheological properties of asphalt are not considered.

**Figure 8:**
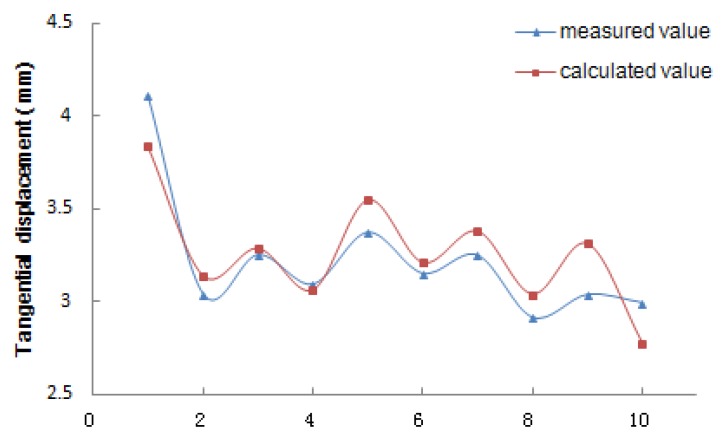
Expressive effect of tangential displacement.

Figure 8 shows that the numerical simulation results were basically consistent with the experimental results and that the displacement obtained by the experiment and the displacement obtained by the software simulation under the interlayer friction coefficient are the same in order of magnitude, and only the specific values are slightly different. The relative accuracy of the software simulation and test analysis is as high as 92% and the friction coefficient of the water-free salt erosion test piece is about 0.85 at 25 °C, while after the bottom-up erosion of the 14% salt solution the friction coefficient is about 0.43. It can be seen that water and salt will have a great impact on the contact state between the base and the surface layer of the road structure.

## 4. Conclusions and Discussions

In this work, both numerical and experimental analyses for predicting the interlayer behavior of specimens were conducted. Firstly, the feasibility of describing interlaminar bonding with the interlaminar shear modulus (K) was analyzed using the Goodman model and four-stage interlaminar shear-stress-displacement model. The contact state between layers of asphalt pavement was measured and evaluated by a 45° inclined shear test under different coupling effects of water and salt entry mode, salt solution concentration, and temperature. The shear test showed that under the same temperature and salt solution concentration, the interlaminar shear modulus values of the three entry modes were top-down > direct erosion > bottom-up. The higher the concentration of the salt solution was, the lower the interlaminar shear modulus was at the same entry mode and temperature of water and salt. For the same concentration of the salt solution, the interlaminar shear modulus decreased with increase in temperature. From bottom to top, the interlaminar shear modulus decreased with change in temperature stress and shrinkage stress of water–temperature materials. From top to bottom, the stiffness modulus of asphalt decreased sharply and the deformation resistance decreased sharply, leading to a decrease in interlaminar shear modulus.

The interlaminar friction coefficient of the specimens with real layers contacting anhydrous salt erosion under test conditions was about 0.85 by finite element simulation, which is much higher than that of any specimens with water salt erosion. The interlayer friction coefficient under 0% salt solution erosion was obviously higher than that under 7% and 14% salt solution erosion and the interlayer friction coefficient decreased with the increase in salt solution concentration, i.e., the higher salt solution concentration, the worse the interlayer bonding state. At the same concentration, the top-down interlayer friction coefficient was the highest, followed by direct erosion, and the bottom-up friction coefficient was the worst. The results of finite element simulation were consistent with those obtained from the characterization of interlaminar shear modulus (K). Combined with the experiment and numerical simulation, it can hence be concluded that water and salt erosion has a great influence on the contact state of the road structure base. This is a significant finding that should be taken into account in standardization and in defining practical requirements.

However, the finite element model should also consider the changing interface properties, which can be represented by cohesive zone models. In order to comprehensively analyze the damage mechanism of asphalt pavement eroded by water and salt, it is necessary to comprehensively understand the water–vapor–salt migration, diffusion, and its spatial and temporal distribution characteristics. On this basis, subgrade structure deformation, pavement material performance decay, and the contact working state change between pavement structure layers caused by water and salt erosion should be comprehensively analyzed so as to realize asphalt control. A dynamic, real, and efficient numerical simulation of mechanical responses of pavement structure has been carried out in this work in order to study the evolution characteristics of the mechanical behavior of asphalt pavement structure. Furthermore, the perception of damage and damage behavior of asphalt pavement eroded by water and salt are put forward.

## Figures and Tables

**Table 1:** Material parameters of specimens.

Layer Position	Modulus of Elasticity/MPa (20 °C)	Thickness (cm)	Poisson Ratio	Density (kg/m^3^)	Damping Value
Asphalt concrete surface	1800	5	0.25	2400	0.9
Cement-stabilized macadam base	2800	5	0.20	2300	0.8

**Table 2:** Comparisons between experimental results and numerical simulation results.

Salt Solution Concentration (%)	Water and Salt Entry Mode	Vertical Force (N)	Vertical Pressure (Pa)	Vertical Displacement of Test (mm)	Equivalent Tangential Displacement (mm)	Calculated Displacements (mm)	Corresponding Interlayer Friction Coefficient
-	-	12,324	770,250	5.810	4.108	3.838	0.85
0	Bottom-up	9028.33	564,270.63	4.300	3.041	3.139	0.57
0	Direct erosion	9939	621,187.5	4.600	3.253	3.288	0.62
0	Top-down	9027.98	564,248.75	4.376	3.094	3.063	0.64
7	Bottom-up	9913.18	619,873.75	4.771	3.374	3.551	0.545
7	Direct erosion	9568	598,000	4.458	3.152	3.211	0.547
7	Top-down	9671.02	60,438.75	4.596	3.250	3.381	0.55
14	Bottom-up	8365.70	522,856.25	4.124	2.914	3.040	0.43
14	Direct erosion	8233.867	514,616.69	4.297	3.038	3.314	0.46
14	Top-down	8704.58	544,036.25	4.234	2.994	2.775	0.46

## References

[1] Chen Y., Hu K., Cao S. (2019). Thermal Performance of Novel Multilayer Cool Coatings for Asphalt Pavements. Materials.

[2] Cui P., Xiao Y., Fang M., Chen Z., Yi M., Li M. (2018). Residual Fatigue Properties of Asphalt Pavement after Long-Term Field Service. Materials.

[3] Jahanbakhsh H., Karimi M.M., Jahangiri B., Nejad F.M. (2018). Induction heating and healing of carbon black modified asphalt concrete under microwave radiation. Constr. Build. Mater..

[4] José R.G., David A.C., Pedro L.C., Miguel Z.P. (2019). Bonding Evaluation of Asphalt Emulsions used as Tack Coats through Shear Testing. Appl. Sci..

[5] Romanoschi S.A., Metcalf J.B. (2001). Characterization of asphalt concrete layer Interfaces. Transp. Res. Rec..

[6] Kruntcheva M.R., Collop A.C., Thom N.H. (2005). Effect of bond condition on flexible pavement performance. J. Transp. Eng..

[7] Uzan J., Livnch M., Eshed Y. (1978). Investigation of adhesion properties between asphaltic-concrete layers. Asph. Paving Technol..

[8] Raab C., Partl M.N. (1998). Shear strength properties between asphalt pavements layers. Arch. Civ. Eng..

[9] Collop A.C., Thom N.H., Sangiorgi C. (2003). Assessment of bond condition using the Leutner shear test. Proc. Inst. Civ. Eng. Transp..

[10] Raposeiras A.C., Vega-Zamanillo A., Calzada-Pérez M., Castro-Fresno D. (2013). New procedure to control the tack coat applied between bituminous pavement layers. Constr. Build. Mater..

[11] Raposeiras A., Castro-Fresno D., Vega-Zamanillo A., Rodriguez-Hernandez J. (2013). Test methods and influential factors for analysis of bonding between bituminous pavement layers. Constr. Build. Mater..

[12] Tashman L., Nam K., Papagiannakis T., Willoughby K., Pierce L., Baker T. (2008). Evaluation of construction practices that influence the bond strength at the interface between pavement layers. J. Perform. Constr. Facil..

[13] West R.C., Moore J.R., Zhang J. Evaluating tack coat applications and the bond strength between pavement layers. Proceedings of the 2006 Airfield and Highway Pavement Specialty Conference.

[14] Liu H., Qiu Y., Jiang X. Experimental evaluation of notch and tack coat that influences the bond strength at the interface between asphalt mixture layers. Proceedings of the ICTE 2013—The 4th International Conference on Transportation Engineering.

[15] Tarr S.M., Okamoto P.A., Sheehan M.J., Packard R.G. (1999). Bond interaction between concrete pavement and lean concrete base. Transp. Res. Rec..

[16] De Carteret R., Watson M., Buzzi O., Fityus S. Effects of dryland salinity on shear and tensile strength of road pavement materials. Proceedings of the 7th International Conference on Maintenance and Rehabilitation of Pavements and Technological Control.

[17] Niu D., Jiang L., Bai M., Miao Y. (2013). Study of the performance of steel fiber reinforced concrete to water and salt freezing condition. Mater. Des..

[18] Yang D., Luo J. (2012). The damage of concrete under flexural loading and salt solution. Constr. Build. Mater..

[19] Hassan Y., Abd El Halim A.O., Razaqpur A.G., Bekheet W., Farha M.H. (2002). Effects of runway deicers on pavement materials and mixes: Comparison with road salt. J. Transp. Eng..

[20] Cui Y., Han J., Li Z., Zhang S., Liu Z. (2015). Research on the performance and microstructure of asphalt under salt freezing cycle. J. Funct. Mater..

[21] Liu Z., Sha A., Xing M., Li Z. (2015). Low temperature property and salt releasing characteristics of antifreeze asphalt concrete under static and dynamic conditions. Cold Reg. Sci. Technol..

[22] Zhang X., Pang L., Wu S., Zhang G. (2018). Effect of different media aqueous solution on road performance of asphalt mixtures. Mater. Sci. Forum.

[23] Ketcheson S.J., Price J.S., Tighe S.L., Stone M. (2014). Transport and retention of water and salt within pervious concrete pavements subjected to freezing and sand application. J. Hydrol. Eng..

[24] Nili M., Zaheri M. (2011). Deicer salt-scaling resistance of non-air-entrained roller-compacted concrete pavements. Constr. Build. Mater..

[25] Hu L., Hu M., Weng Y. (2005). The test methods of road layer adhesion strength. Shanxi Sci. Technol. Commun..

[26] Ma C. (2017). Research on Influence of Chloride Deicing Salt’s Concentration on Performances of Asphalt and Mixture. Master’s Thesis.

[27] Li P., Nian T., Zhang Y. (2015). Study on Anti-shearing Property of Asphalt Pavement for Concrete Bridge Deck Based on Oblique Shear Test. J. Wuhan Univ. Technol..

[28] Wang R., Yu M. (2009). Application of Inclined Plane Shear Test to Asphalt Mixture. J. Chongqing Jiaotong Univ. (Nat. Sci.).

[29] Yildirim Y., Smit A.D.F., Korkmaz A. (2005). Development of a laboratory test procedure to evaluate tack coat performance. Turk. J. Eng. Environ. Sci..

[30] Zhao Y. (2012). Mechanical Properties and Engineering Application of Cement Stabilized Granules Containing RAP. Master’s Thesis.

[31] Lan H. (2011). Research on Water Damage Behavior and Mechanism of Asphalt Pavement. Master’s Thesis.

[32] Zhou Z. (2009). Research on the Application Under-Seal Synchronous Pavement. Master’s Thesis.

